# Sustained-release solid dispersion of pelubiprofen using the blended mixture of aminoclay and pH independent polymers: preparation and *in vitro*/*in vivo* characterization

**DOI:** 10.1080/10717544.2017.1399304

**Published:** 2017-11-10

**Authors:** Yeo-Song Lee, Jae Guen Song, Sang Hoon Lee, Hyo-Kyung Han

**Affiliations:** College of Pharmacy, Dongguk University-Seoul, Goyang, Korea

**Keywords:** Pelubiprofen, sustained-release, solid dispersion, dissolution, aminoclay, Eudragit® RL PO, Eudragit® RS PO

## Abstract

The present study aimed to develop the sustained-release oral dosage form of pelubiprofen (PEL) by using the blended mixture of 3-aminopropyl functionalized-magnesium phyllosilicate (aminoclay) and pH-independent polymers. The sustained-release solid dispersion (SRSD) was prepared by the solvent evaporation method and the optimal composition of SRSD was determined as the weight ratio of drug: Eudragit® RL PO: Eudragit® RS PO of 1:1:2 in the presence of 1% of aminoclay (SRSD(F6)). The dissolution profiles of SRSD(F6) were examined at different pHs and in the simulated intestinal fluids. The drug release from SRSD(F6) was limited at pH 1.2 and gradually increased at pH 6.8, resulting in the best fit to Higuchi equation. The sustained drug release from SRSD(F6) was also maintained in simulated intestinal fluid at fasted-state (FaSSIF) and fed-state (FeSSIF). The structural characteristics of SRSD(F6) were examined by using powder X-ray diffraction (PXRD), differential scanning calorimetry (DSC) and Fourier transform infrared spectroscopy (FT-IR), indicating the change of drug crystallinity to an amorphous form. After oral administration in rats, SRSD(F6) exhibited the prolonged drug exposure in plasma. For both PEL and PEL-transOH (active metabolite), once a day dosing of SRSD(F6) achieved oral exposure (AUC) comparable to those from the multiple dosing (3 times a day) of untreated drug. In addition, the *in vivo* absorption of SRSD(F6) was well-correlated with the *in vitro* dissolution data, establishing a good level A *in vitro/in vivo* correlation. These results suggest that SRSD(F6) should be promising for the sustained-release of PEL, thereby reducing the dosing frequency.

## Introduction

Pelubiprofen (PEL) (2-[4-(2-oxocyclohexylidenemethyl) phenyl] propionic acid) is a nonsteroidal anti-inflammatory drug (NSAID) and its pharmacological effect is mediated by the multiple pathways including inhibition of cyclooxygenases (COX) activity, TAK1-IKK-NF-κB pathway and prostaglandin synthesis (Shin et al., 2011). PEL is effective against rheumatoid arthritis, osteoarthritis, and pain relief (Shin et al., 2011). Oral absorption of PEL is very fast, where T_max_ of PEL is within 15 min in rats and 1 h in human (Takasaki et al., 1993; Song et al., 2016). In addition, PEL is metabolized rapidly to unsaturated-, cis-, and trans-alcohols after the intestinal absorption (Asami et al., 1996; Ryu et al., 2017). The plasma half-lives of PEL and its active metabolite (PEL-transOH) are 0.36 h and 1.5 h, respectively (Matsuki et al., 1993). Therefore, the frequent dosing of PEL is required to maintain the adequate plasma levels.

Sustained-release (SR) dosage forms can reduce the dosing frequency by maintaining therapeutically effective drug concentrations over a prolonged period of time and improve the patient compliance (Haan & Lerk, 1984; Khodaverdi et al., 2017). Furthermore, a properly designed SR formulation can provide some additional benefits including low cost, improved efficacy, and reduced adverse effects (Colombo et al., 2000). Among various formulation approaches for sustained drug release, preparation of matrices with insoluble polymers using solid dispersion technique is an effective way to produce the SR oral dosage forms. In the polymer matrix systems, the drug molecule is homogeneously distributed throughout a matrix and drug release rate is controlled by water-swellable or erodible matrices consisting of various hydrophilic or hydrophobic polymeric excipients (Kim et al., 2016; Son et al., 2017). Time dependent and pH-independent polymers such as ethyl cellulose (EC), hydroxypropyl cellulose (HPC), Eudragit® RL and Eudragit® RS are often employed to prepare the appropriate sustained-release matrix formulations. In particular, methacrylic resins (Eudragit®) are commonly used as matrix forming polymers of SRSD, since they exhibit high chemical stability, compactability, and variability in physicochemical characteristics (Wadher et al., 2011). Among methacrylic resins, Eudragit® RS PO is a polymer synthesized from acrylic and methacrylic acid esters, containing low-level quaternary ammonium groups, whereas Eudragit® RL PO contains higher level of quaternary ammonium groups (Pignatello et al., 2002; Pawar & Gautam, 2016). Both of them are insoluble at physiological pH but capable of swelling. Therefore, Eudragit® RL PO and Eudragit® RS PO are selected to provide the polymeric matrix of SRSD formulations in the present study. When polymers are used in combination, there might be the synergistic effect in improving the stability and dissolution of amorphous drugs in solid dispersion systems (Prasad et al., 2014; Baghel et al., 2016; Ziaee et al., 2017). For example, Prasad et al. (2014) have developed the ternary solid dispersion of indomethacin with Eudragit E100 and PVP K90, which exhibited higher stability and dissolution than binary dispersions. They also demonstrated that the combination of polymers could lead to greater drug-polymer miscibility in ternary dispersions than binary dispersions.

3-Aminopropyl functionalized magnesium phyllosilicate (aminoclay) is also included as a pH modifier in SRSD formulation since PEL is an acidic drug having a good solubility in basic pHs (Song et al., 2016; Yamashita et al., 2017). Aminoclay is delaminated to cationic and water soluble nanosheets in water. Aminoclay can enhance the solubility of acidic drugs by modulating the microenvironmental pH to more basic (Yang et al., 2013; Yang et al., 2016). In addition, aminoclay can control the drug release by interacting with negatively charged drug and/or polymers (Holmström et al., 2007). Aminoclay is biocompatible and is readily eliminated through the urine and feces without any long-term accumulation in tissues (Yang et al., 2014a).

Given that the combination of polymers have the synergistic effect in enhancing the stability and dissolution of amorphous drugs in solid dispersion systems and the incorporation of pH modifiers can alter the microenvironmental pH within and surrounding drugs, thus improving the dissolution of weakly acidic drugs, the appropriate organic-inorganic combinations of aminoclay and pH-independent polymers having high and low permeability may be suitable for sustaining the drug release and assure more reproducible drug release behavior. Therefore, the present study aimed to (i) develop SRSD formulation of PEL by using blended mixture of aminoclay, Eudragit® RL PO and Eudragit® RS PO and (ii) evaluate their *in vitro* and *in vivo* characteristics.

## Materials and methods

### Materials

PEL was kindly provided by Daewon Pharm. Co., Ltd. (Seoul, Korea). Eudragit® RL PO and Eudragit® RS PO were obtained from Evonik Korea Ltd. (Seoul, Korea). 3-Aminopropyltriethoxysaline (ATPES, 99%), sodium taurodeoxycholate (NaTDC), and tolbutamide were purchased from Sigma-Aldrich Co. (St. Louis, MO, USA). Lecithin (ca. 99.2% soy bean phosphatidylcholine) was purchased from Avanti lipid (USA). Magnesium chloride hexahydrate (98%) and other inorganic salts were obtained from Junsei Chemical Co., Ltd. (Tokyo, Japan). Aminoclay was prepared by the method reported by Yang et al. (2013). Acetone was purchased from Daejung chemical & metals Co., Ltd. (Gyeonggi-Do, Korea). All other chemicals were of analytical grade and all solvents were of high performance liquid chromatography (HPLC) grade.

### Preparation of SRSD formulations

SRSDs were prepared by solvent evaporation method. PEL, Eudragit® RL PO and Eudragit® RS PO were dissolved in acetone. Then, aminoclay suspended in acetone was added to the solution of drug and polymers. After vigorous mixing, the solvents were evaporated at room temperature and dried completely in a vacuum oven for 24 h. The obtained SRSDs were milled and passed through a mesh 45 sieve. Physical mixtures (PMs) were obtained by mixing crystalline PEL, polymers, and aminoclay in a mortar and pestle. For the optimization of compositions, SRSDs were prepared at the various weight ratios of each component (drug, Eudragit® RL PO, Eudragit® RS PO, aminoclay) and denoted as formulations F1-F7, respectively. The formulation details are summarized in Table 1.

**Table 1.** Composition of pelubiprofen loaded-solid dispersions.

	Ratio (w/w/w)			
Formulation	Pelubiprofen	Eudragit® RL PO	Eudragit® RS PO	Aminoclay (%)
F1	1	1	8	5
F2	1	1	6	5
F3	1	1	2	5
F4	1	2	2	5
F5	1	0	2	5
F6	1	1	2	1
F7	1	1	2	0

### *In vitro* dissolution study

Dissolution studies were performed in a dissolution tester DT 1420 (ERWEKA, Heusenstamm, Germany) using the US Pharmacopeia (USP) paddle method at 37 ± 0.5 °C and 50 rpm. Each formulation contained drug amount equivalent to 30 mg of PEL and was filled in hard gelatin capsules with 5% L-HPC (low-substituted HPC) as a disintegrant. Each formulation (F1-F7) was exposed to the dissolution medium at pH 6.8 for 12 h. One ml of each sample was withdrawn at the predetermined time points (0.25, 0.5, 0.75, 1, 1.5, 2, 4, 6, 8, and 12 h) and the equal volume of fresh medium was added into the vessel to maintain the constant volume of dissolution media. The collected samples were filtered through 0.45 µm pore-sized syringe filters. The filtrates were diluted with the mobile phase and the released drug amount was analyzed by an HPLC assay.

Once the optimal SRSD formulation (F6) was selected, its drug release profiles were also evaluated at different pHs (pH 1.2, 4.0, 6.8, and water). Furthermore, in order to simulate the pH change along the gastrointestinal tract, the *in vitro* drug release characteristics of the optimal SRSD formulation (F6) were evaluated by the buffer transition method and compared with those from untreated drug and its corresponding PM. SRSD formulation (F6) was exposed to acidic condition (pH 1.2) for initial 2 h and then dissolution media was replaced with pH 6.8 phosphate buffer for 10 h. In the acidic stage, each formulation was exposed to 750 mL of 0.1 N hydrochloric acid solution for 2 h and then 250 mL of 0.2 M tribasic sodium phosphate solution was added into the vessels for the transition to the buffer at pH 6.8. Drug release assessment was continued for next 10 h. At predetermined time points (0.25, 0.5, 1, 1.5, 2, 2.5, 3, 3.5, 4, 5, 6, 8, and 12 h), 1 mL of each sample was collected and the equal volume of fresh medium was added into the vessel. The collected samples were filtered through 0.45 µm pore-sized syringe filters. After the adequate dilution of the filtrates with the mobile phase, the released drug amount was analyzed by an HPLC assay.

### Drug release studies in the simulated intestinal fluids

Drug release profiles of SRSD formulation were examined in fasted-state simulated intestinal fluid (FaSSIF) and fed-state simulated intestinal fluid (FeSSIF) by using the USP paddle method at 37 ± 0.5 °C and 50 rpm. FaSSIF and FeSSIF were prepared as described by the previous reports (Jantratid et al., 2008; Marques et al., 2011). FaSSIF was composed of 3 mM sodium taurocholate, 0.2 mM lecithin, 19.12 mM maleic acid, 34.8 mM sodium hydroxide and 68.62 mM sodium chloride. FeSSIF was composed of 10 mM sodium taurocholate, 3 mM lecithin, 28.6 mM maleic acid, 52.5 mM sodium hydroxide, 145.2 mM sodium chloride, 6.5 mM glyceryl monocholate and 40 mM sodium oleate. The pH of FaSSIF and FeSSIF were adjusted to 6.5. Each formulation (SRSD (F6), untreated drug, PM) was exposed to FaSSIF and FeSSIF, respectively for 12 h. The samples were withdrawn at 0.25, 0.5, 1, 1.5, 2, 3, 4, 6, 8, and 12 h, followed by the addition of an equal volume of fresh medium to the vessel. The collected samples were filtered through 0.45 µm pore-sized syringe filters. All filtrates were diluted appropriately with mobile phase and analyzed by an HPLC assay.

### Drug release kinetics

Drug release kinetics were evaluated by applying the dissolution data to the different kinetic equations such as zero order (Equation (1)), first order (Equation (2)) (Costa & Lobo, 2001), Higuchi (Equation (3)) (Higuchi, 1963), Hixson-Crowell (Equation (4)) (Hixson & Crowell, 1931) and Korsmeyer-Peppas (Equation (5)) (Korsmeyer et al., 1983) equations.

(1) $${\text{Q}}_{\text{t}}={\text{k}}_{0}\text{t}$$

(2) $$\ln\;\left({\text{Q}}_{0}-{\text{Q}}_{\text{t}}\right)=-{\text{k}}_{\text{1}}\text{t}+\ln\;{\text{Q}}_{0}$$

(3) $${\text{Q}}_{\text{t}}={\text{k}}_{\text{2}}{\text{t}}^{\text{1}/\text{2}}$$

(4) $${\left(\text{1}-{\text{Q}}_{\text{t}}\right)}^{\text{1}/\text{3}}=\text{1}-{\text{k}}_{\text{3}}\text{t}$$

(5) $${\text{M}}_{\text{t}}/{\text{M}}_{\infty }={\text{k}}_{\text{4}}{\text{t}}^{\text{n}}$$

Where Q_0_ is the initial amount of drug, Q_t_ is the released drug amount at time t and k_0,_ k_1_, k_2_, k_3_, and k_4_ are the release rate constant of zero order, first order, Higuchi, Hixson-Crowell and Korsmeyer-Peppas model, respectively. M_t_/M_∞_ is the fraction of released drug at time t and n is the parameter depending on the release mechanism. Each parameter was determined by linear least-squares fitting methods and best fits were assessed by the correlation factors as *r*^2^.

### Differential scanning calorimetry (DSC)

Thermal transition properties of samples were determined using a DSC Q2000 (TA Instruments, Ghent, Belgium) equipped with an intercooler. Indium was used to calibrate the temperature and heat flow. Samples were accurately weighed (5–15 mg) and placed in aluminum pans. The thermograms were obtained at a scanning rate of 10 °C/min over a temperature range of 20–150 °C under an inert atmosphere flushed with nitrogen at a rate of 30 mL/min.

### Powder X-ray diffraction (PXRD)

PXRD was performed at room by using an X-ray diffractometer (X’Pert APD, PHILIPS, Amsterdam, Netherlands). The diffraction pattern was measured with CuKα radiation at 40 kV and 30 mA over a 2*θ* range of 3–40° using a step size of 0.02° at a scan speed of 1 s/step. The samples were placed on a zero-background silicon holder. XRD experiments were conducted at the Korea Basic Science Institute (Daegu Center, Korea).

### Fourier transform infrared spectroscopy (FT-IR)

FT-IR spectra of samples were obtained by the ATR-FT-IR spectroscopy (Nicolet™ iS™ 5; Thermo-Fisher Scientific, Waltham, MA, USA) with a ZnSe crystal accessory. Each spectrum was obtained over the wavelength range of 4000-600 cm^−1^ with 32 scans at a resolution of 4 cm^−1^.

### Pharmacokinetic studies in rats

Animal studies were carried out in accordance with the “Guiding Principles in the Use of Animals in Toxicology” adopted by the Society of Toxicology (USA) and the study protocol was approved by the review committee of Dongguk University (IACUC-2016-038-1). Male Sprague-Dawley rats (250–290 g) were supplied by Orient bio Co., Ltd. (Seongnam, Korea). All rats were given free access to tap water and a normal standard chow diet (Superfeed Company, Wonju, Korea). Prior to the experiments, rats were fasted for 12 h and divided into two groups (*n* = 3 per group). Untreated drug (3 mg/kg of PEL) was given orally three times at 0, 8, and 16 h and SRSD (F6) was given orally once at the dose equivalent to 9 mg/kg of PEL. Blood samples were collected from the femoral artery at the predetermined time points. Blood samples were centrifuged at 13,000 rpm for 5 min and the obtained plasma samples were kept frozen at −80 °C until analyzed by HPLC.

#### HPLC assay

Drug concentrations of *in vitro* samples were determined by using an HPLC system (Perkin Elmer series 200) and a reversed-phase C18 column (Gemini C18, 4.6 × 250 mm, 5 μm; Phenomenex, Torrance, CA, USA). The mobile phase consisted of acetonitrile and 0.1% formic acid (65:35, v/v) and the flow rate was 1 mL/min at 30 °C. The injection volume was 10 μL and the UV wavelength set at 225 nm. Ibuprofen was used as an internal standard. The calibration curve of PEL was linear within the concentration range of 0.5–40 μg/mL.

#### LC-MS/MS assay

Plasma concentrations of PEL and PEL-transOH were determined by using LC–MS/MS. Chromatographic separation was done with a C18 column (4.6 × 100 mm, 2.6 μm; Phenomenex, Torrance, CA, USA) at 40 °C. Mobile phase for PEL consisted of solvent A (0.1% acetic acid) and solvent B (acetonitrile), with a gradient elution (0–9 min, 30%–70%; 9–10 min, 30% of solvent A) and the flow rate was 0.5 mL/min. For PEL-transOH, isocratic elution was used with 0.1% acetic acid and acetonitrile (35:65, v/v) at a flow rate of 0.2 mL/min. Mass spectrometric detection was done by an AB Sciex API 4000 triple quadrupole mass spectrometer (AB Sciex, Framingham, MA, USA). The electrospray ionization (ESI) source was set in positive ionization mode for PEL and in negative ionization mode for PEL-transOH. The precursor/product ion pair (m/z) was 259.1/195.3 for PEL, 260.3/216.8 for PEL-transOH, and 271.1/155 for tolbutamide (IS). The calibration curves for PEL and PEL-transOH were prepared at the concentrations of 1–100 ng/mL and 50-8000 ng/mL, respectively, and showed a good linearity with a *r*^2^ value greater than 0.99.

### Pharmacokinetic data analysis and *in vitro/in vivo* correlation (IVIVC)

Noncompartmental analysis was performed using WinNonlin® (Pharsight Co., Sunnyvale, CA, USA). The maximum plasma concentration (C_max_) and the time to reach the maximum plasma concentration (T_max_) were observed values from the plasma concentration-time profiles. The area under the plasma concentration–time curve (AUC) was calculated by the linear trapezoidal method.

An IVIVC was evaluated by plotting the percent drug released *in vitro* versus the percent drug absorbed (*Fa*) *in vivo*. Percent drug released data were obtained from *in vitro* dissolution studies and the percent drug absorbed data were determined at various time points by Wagner–Nelson method using the following equation (Wagner, 1983):

$$F_{a}(\%)=\frac{C_{t}+k_{e}{AUC}_{\mathrm{0-}t}}{k_{e}{AUC}_{0-\infty }}\times 100$$

where *Fa* is the fraction dose absorbed, *C_t_* is the drug concentration in plasma at time *t*, *k_e_* is the elimination rate constant, AUC_0–_*_t_*, and AUC_0–∞_ are areas under the curve from time zero to time *t* and to infinity, respectively. Linear regression analysis was applied to fit the data and *r*^2^ was calculated to evaluate the robustness of IVIVC.

### Statistical analysis

All the data are expressed as mean ± standard deviation (SD). Statistical analysis was performed using Student’s *t*-test or one-way ANOVA followed by Dunnett’s correction. A *p* value <.05 was considered statistically significant.

## Results and discussion

### Preparation of SRSD and optimization of formulation composition

**Figure 1.**
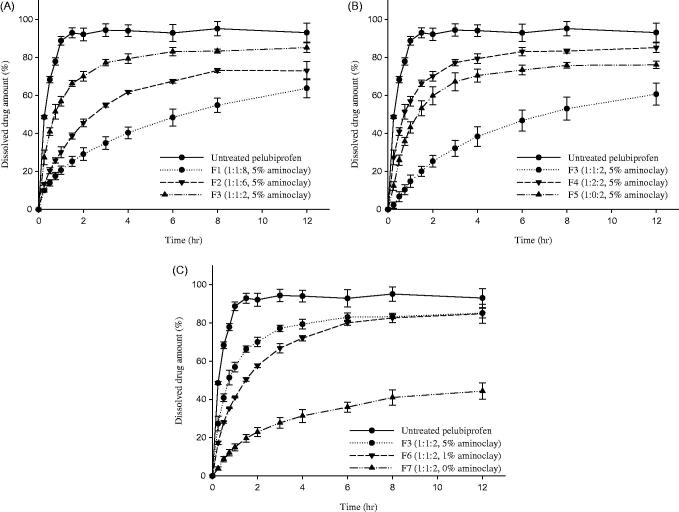
Dissolution profiles of PEL from different formulations (F1-F7) at pH 6.8 (Mean ± SD, *n* = 3). (A) Effect of Eudragit® RS PO, (B) effect of Eudragit® RL PO, (C) effect of aminoclay.

To optimize the drug-excipients ratios, various SRSD formulations (F1-F7) were prepared as summarized in Table 1 and the dissolution studies were carried out at pH 6.8. As illustrated in Figure 1, all the tested SRSD formulations exhibited slower drug release, depending on the ratio of Eudragit® RL PO and Eudragit® RS PO, while untreated drug was almost completely dissolved within 1 h. Both Eudragit® RL PO and Eudragit® RS PO are insoluble at physiological pH but capable of swelling after solvent penetration (Wulff & Leopold, 2014). Therefore, as exposed to an aqueous medium, those polymer matrix forms a highly viscous gelatinous barrier, controlling the drug release as well as the penetration of dissolution medium into the matrix (Reza et al., 2002). In particular, the dissolution rate was gradually delayed as the amount of Eudragit® RS PO in the matrix was increased (Figure 1(A)). When the ratio of Eudragit® RL PO and Eudragit® RS PO increased from 1:2 (F3) to 1:8 (F1), the released drug amount decreased from 70% to 29% after 2 h and from 85% to 63% after 12 h. Given that Eudragit® RS PO has low permeability in the dissolution media as compared with Eudragit® RL PO, it may act as a strong release retardant polymer, delaying the penetration of dissolution medium into the matrix and decreasing the drug release rate (Boyapally et al., 2009). Accordingly, when Eudragit® RS PO was used as the only matrix forming polymer, drug release rate was too slow and the released drug amount of F5 was only 60% in 12 h (Figure 1(B)). In contrast, the addition of Eudragit® RL PO significantly enhanced the drug dissolution. Since the higher level of quaternary ammonium groups makes Eudragit® RL PO more hydrophilic and more permeable than Eudragit® RS PO (Abbaspour et al., 2005), the amount of the present quaternary ammonium groups and the water permeability can be adjusted by blending Eudragit® RL PO and Eudragit^®^ RS PO. Hence, the combined use of Eudragit^®^ RL PO and Eudragit^®^ RS PO in SRSD formulations achieved more desirable drug release profiles than SRSD using only Eudragit^®^ RS PO (Figure 1B). Previous studies also have observed that the combination of polymers had the synergistic effect in enhancing the stability and dissolution of amorphous drugs in ternary dispersion systems (Prasad et al., 2014; Baghel et al., 2016; Ziaee et al., 2017).

On the other hand, presence of aminoclay significantly enhanced the drug release rate. As shown in Figure 1(C), F7 formulation lacking aminoclay exhibited very slow drug release with about 44% of dissolved drug amount after 12 h, while F3 formulation containing 5% of aminoclay achieved approximately 57% of drug dissolution within 1 h indicating initial burst release. Aminoclay can drive the microenvironmental pH within and surrounding a dissolving solid to more basic, thereby making PEL, an acidic drug (p*K*_a_ = 4.6) more soluble (Tran et al., 2008; Yang et al., 2014b). Therefore, diffusional exchange of dissolved drug between the polymeric matrix and the bulk dissolution medium may be increased. Moreover, the potential ionic interactions between the carboxylic acid group of PEL and the amine group of aminoclay may help the dissolution of PEL.

Overall, among the tested formulations, F6 formulation exhibited desirable sustained drug release profiles, where the released drug amount was 41% in 1 h and 85% in 12 h. Therefore, F6 formulation was selected as an optimal SRSD formulation for PEL and further characterization was focused on F6 formulation (SRSD (F6)).

**Figure 2.**
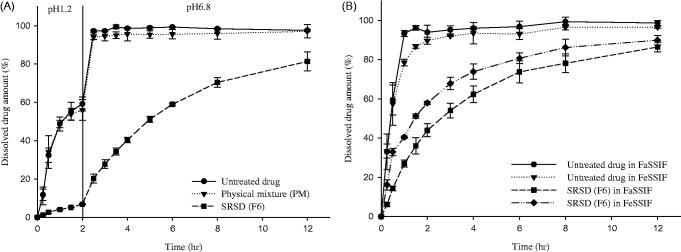
Dissolution profiles of PEL from SRSD (F6) in the different dissolution media (Mean ± SD, *n* = 3). (A): in buffer transition system, (B): in FaSSIF and FeSSIF.

In order to examine the pH-dependency in drug release, the dissolution profiles of SRSD (F6) were evaluated at the different pHs. The dissolution of SRSD (F6) was very limited in acidic condition (pH 1.2 and pH 4.0), indicating approximately 20%-30% of drug dissolution for 12 h (*See supplementary data, S1*). However, it achieved 85% of drug dissolution at pH 6.8. Similar findings were observed from the drug release studies in buffer transition system. To mimic the intestinal pH gradient, dissolution studies of SRSD (F6) were also performed by using buffer transition systems and compared with those from untreated drug and PM. Assuming maximum GI tract transit time of 12 h and gastric emptying time of generally 2 h (Rawat et al., 2007; Vasavid et al., 2014), dissolution studies of SRSD (F6) was carried out at the gastric pH (pH 1.2) for 2 h and the intestinal pH (pH 6.8) for 10 h. As illustrated in Figure 2(A), while the untreated drug and PM showed fast dissolution, SRSD (F6) showed a drug release less than 7% at pH 1.2 for 2 h. After transition to the intestinal stage at pH 6.8, drug release from SRSD (F6) gradually increased, suggesting that SRSD (F6) may exhibit sustained drug release in GI tract.

The pH dependency in dissolution of SRSD (F6) may be explained by the pH dependent solubility of PEL since PEL exhibits low solubility at acidic condition (Song et al., 2016). In addition, it may be also attributed to the difference in the hydration and permeability of polymer matrix in different dissolution media. Quaternary ammonium groups of Eudragit^®^ RL PO and Eudragit^®^ RS PO are in the chloride salt form and anions in dissolution media have different capabilities in interacting with the quaternary ammonium groups, thereby influencing the permeability of the polymers. Therefore, drug release from the polymer matrix depends on the exchange between the anions dissolved in the dissolution media and the quaternary ammonium groups of polymer (Wagner & McGinity, 2002). Since the dissolution media at pH 1.2 had chloride anions which are less selective to ion exchange than phosphate ion at pH 6.8, the degree of hydration and permeability of polymer matrix decrease at acidic condition (Bodmeier et al., 1996; Wagner & McGinity, 2002). Some of previous studies also reported that drug release rates from pellets coated with ammonio methacrylate copolymers varies in the dissolution media containing different anions (Knop, 1996; Wagner & McGinity, 2002). For example, Wagner and McGinity (2002) reported that high sodium chloride concentrations decreased the permeability of polymer films by reducing ion exchange. Wulff & Leopold (2014) also reported that higher concentrations of sodium chloride decrease the theophylline release rate from Eudragit^®^ RL/L55 blends.

### Dissolution studies in simulated intestinal fluid under the fasted and fed conditions

PEL is usually administered orally and food intake can affect its *in vivo* dissolution. Therefore, to predict the food effect on *in vivo* drug dissolution, biologically relevant dissolution media was used for *in vitro* simulation of fasted and fed state (Klein, 2010). As summarized in Figure 2(B), the dissolution profiles of untreated drug and SRSD (F6) were examined in FaSSIF and FeSSIF. While untreated drug exhibited fast and almost complete dissolution within 2 h, SRSD (F6) maintained the sustained drug release in both FaSSIF and FeSSIF. Furthermore, in each formulation, the dissolution profiles were similar between FaSSIF and FeSSIF, suggesting that drug dissolution from SRSD (F6) may not be affected by food intake.

### Dissolution kinetics

To examine the drug release kinetics, the dissolution data of SRSD (F6) at pH 6.8 were fitted to various mathematical models including zero order, first order, Higuchi, Hixson-Crowell and Korsmeyer-Peppas models. The regression parameters such as rate constants and the correlation coefficients (*r*^2^) were estimated (*See supplementary data, S2*). The goodness of fit for various models was ranked in order of Higuchi > Korsmeyer–Peppas > first order > Hixson–Crowell > zero order. Accordingly, *in vitro* release data of SRSD (F6) was best fitted to Higuchi equation with the *r*^2^ value of 0.9489, suggesting that the drug release from SRSD (F6) might be governed by the diffusion process. The data were also fitted to Korsmeyer–Peppas equation. As shown in S2 (*See supplementary data)*, SRSD (F6) exhibited good linearity with the release exponent (n) value of 0.4274. These results indicated that the drug release from SRSD (F6) were controlled by Fickian diffusion (Chime et al., 2013; Patil et al., 2015).

### Structural analysis

**Figure 3.**
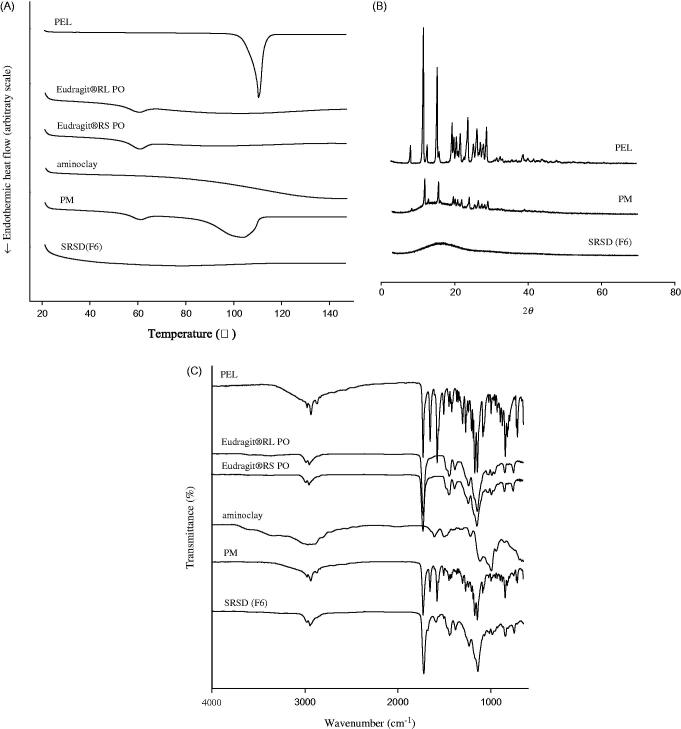
Structural characteristics of SRSD (F6). DSC thermograms (A), PXRD patterns (B), and FT-IR spectra (C).

The thermograms of untreated drug, Eudragit® RL PO, Eudragit® RS PO, aminoclay, PM and SRSD (F6) were examined by DSC. As illustrated in Figure 3(A), the endothermic peak of untreated drug was obtained as 110.38 °C, which was broadened and shifted to 103.82 °C in PM. This might be because PEL in PM was miscible with polymer in the molten state (Paradkar et al., 2004; Gupta et al., 2015). In SRSD (F6) formulation, endothermic drug peak was disappeared, implying the change of drug crystallinity to amorphous state.

X-ray diffraction was used to identify the crystallinity of drug in PM and SRSD (F6). As shown in Figure 3(B), PXRD patterns from untreated drug exhibited many distinct peaks at 2*θ* angles in the range of 8–30°. The characteristic peaks of PEL were retained in PM, indicating that drugs were in a crystalline form. The lower intensity of drug peaks in PM was due to the dilution effect by excipients compared to that from untreated drug. On the other hand, SRSD (F6) showed the absence of distinct diffraction peak of PEL, indicating that drug dispersed into the polymer matrix might be in an amorphous form.

The FT-IR spectra of untreated drug, Eudragit® RL PO, Eudragit® RS PO, aminoclay, PM and SRSD (F6) were examined and illustrated in Figure 3(C). The spectrum of untreated drug showed distinct absorption bands at 1653.09 cm^−1^ and 1728.86 cm^−1^ for the C = O group from carboxylic acid and cyclic ketone, respectively and C = C group from benzene ring at 1576.79 cm^−1^. In PM, the characteristic absorption peaks of drug and excipients were retained, implying that there was no significant interaction between drug and excipients. In the case of SRSD (F6), the characteristic absorption band at carbonyl group (1653.09 cm^−1^) in PEL was disappeared and new peak was observed at 1591.87 cm^−1^, implying that there may be potential interactions of drug-polymers and/or drug-aminoclay.

### Pharmacokinetic studies

**Figure 4.**
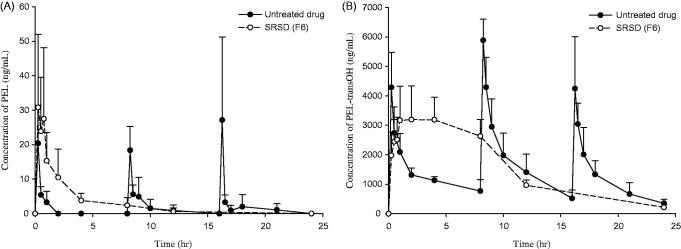
Pharmacokinetic profiles of PEL and PEL-transOH after oral administration of PEL in different formulations to rats ((Mean ± SD, *n* = 3). Untreated drug (3 mg/kg) was given orally three times and SRSD (F6) was given orally once at the dose equivalent to 9 mg/kg of PEL.

The plasma concentration-time profiles of PEL and PEL-transOH are shown in the Figure 4 and pharmacokinetic parameters are summarized in the Table 2. After an oral administration of untreated drug, both PEL and its active metabolite (PEL-transOH) exhibited the fast drug absorption with C_max_ of 30.5 ng/mL and 5886.2 ng/mL within 0.25 h, respectively. Due to the rapid metabolism, the oral exposure of PEL was negligible compared to that of PEL-transOH. On the other hand, the oral administration of SRSD (F6) exhibited a relatively slow drug absorption and prolonged the plasma exposure of PEL-transOH for a longer period of time with significantly (*p* < .05) delayed T_max_. As a result, once a day dosing of SRSD (F6) achieved oral exposure (AUC) of PEL and PEL-transOH comparable to those from the multiple dosing (3 times a day) of untreated drug. These results suggest that SRSD (F6) could sustain the drug release *in vivo* and prolong the systemic exposure of drug, which may lead to improve the therapeutic efficacy and decrease the dosing frequency of PEL.

**Table 2.** Pharmacokinetic parameter of PEL and PEL-transOH after oral administration of PEL in different formulations to rats ((Mean ± SD, *n* = 3).

	PEL		PEL-transOH	
Pharmacokinetics parameters	Untreated drug	SRSD (F6)	Untreated drug	SRSD (F6)
C_max_ (ng/mL)	30.5 ± 21.1	30.9 ± 21.1	5886.2 ± 717.32	3380.2 ± 983.73*
T_max_ (h)	0.25	0.42 ± 0.29	0.25	2.7 ± 1.2*
AUC (ng/mL)	40.7 ± 21.7	62.2 ± 37.9	33998 ± 9603.5	37299 ± 8759.6

* *p* < .05, Compared to untreated drug.

### IVIVC

Released drug amount (%) were obtained from *in vitro* dissolution studies at pH 6.8 and the fraction of the drug absorbed was calculated by using *in vivo* rat PK data as described in method section. Linear regression analysis was performed to fit the data and the correlation coefficient (*r^2^*) was calculated to evaluate IVIVC. A good linear correlation was obtained with the *r*^2^ value of 0.9555 when the fraction of the drug absorbed for PEL-transOH was plotted against the released drug amount (%) *in vitro* (*See supplementary data, S3*). These findings suggest that a close correlation can be established between *in vitro* dissolution of SRSD (F6) and its *in vivo* drug absorption, thereby utilizing *in vitro* data as a surrogate to predict *in vivo* pharmacokinetic behavior (the rate and extent of oral drug absorption) of each formulation. In addition, these results indicated that the dissolution process was the rate-limiting step during the drug absorption in rats.

## Conclusion

The SR solid dispersion of PEL (SRSD (F6)) was prepared by using blended mixture of Eudragit® RL PO, Eudragit® RS PO and aminoclay. The developed SRSD (F6) exhibited the dissolution profiles suitable for sustained drug release, well fitted to Higuchi equation indicating the diffusion controlled drug release. After an oral administration, SRSD (F6) achieved the sustained drug exposure in rats. Furthermore, *in vivo* absorption of SRSD (F6) was well-correlated with *in vitro* dissolution data, establishing a good level A IVIVC. Taken together, SRSD (F6) formulation appeared to be promising for the SR of PEL, potentially reducing the dosing frequency.

## Supplementary Material

Hyo-Kyung_Han_et_al_supplemental_content.zip
