# Supplementary material for: Sustained-release solid dispersion of pelubiprofen using the blended mixture of aminoclay and pH independent polymers: preparation and in vitro/in vivo characterization
Source: Drug Deliv. 2017 Nov 10;24(1):1731–9. doi: 10.1080/10717544.2017.1399304 (PMC8240988; doi:10.1080/10717544.2017.1399304)
Supplement: Hyo-Kyung_Han_et_al_supplemental_content.zip [file IDRD_A_1399304_SM0605.zip › Hyo-Kyung Han et al supplemental content.docx]

**S 1.** Dissolution profiles of PEL from SRSD (F6) at different pHs (Mean±SD, n=3).

**S2.** Release kinetic parameters of pelubiprofen in SRSD formulation

| Formulation | Zero order | |  | First order | |  | Higuchi model | | Hixson-Crowell | | |  | Korsmeyer-Peppas | | |
| --- | --- | --- | --- | --- | --- | --- | --- | --- | --- | --- | --- | --- | --- | --- | --- |
|  | r^2^ | k_1_ |  | r^2^ | k_2_ |  | r^2^ | k_3_ |  | r^2^ | k_4_ |  | r^2^ | k_5_ | n |
| SRSD (F6) | 0.7635 | 9.0136 |  | 0.9252 | 0.2151 |  | 0.9489 | 30.416 |  | 0.879 | 0.244 |  | 0.9451 | 38.3089 | 0.4274 |

r^2^, correlation coefficient; k_1 –_ k_5_, constants of release kinetics; n, diffusional exponent

**S3.** IVIVC of SRSD (F6) formulation
